# Contrast-enhanced ultrasound for the evaluation of CXCR7-mediated angiogenesis in colon cancer

**DOI:** 10.7150/jca.82438

**Published:** 2023-03-05

**Authors:** Xiang Li, Zitao Li, Caijuan Li, Xuemei Wang, Bin Jiang, Jiannan Wang, Zhen Zhang

**Affiliations:** 1 Department of Ultrasonic Diagnosis, The First Hospital of China Medical University, 155 Nanjing North Street, Shenyang 110001, Liaoning, PR China; 2 Department of Orthopedic Surgery, Hongqi Hospital, Mudanjiang Medical University, 5 Tongxiang Road, Mudanjiang, Heilongjiang 157011, PR China; 3 Department of Ultrasound, Hongqi Hospital, Mudanjiang Medical University, 5 Tongxiang Road, Mudanjiang, Heilongjiang 157011, PR China

**Keywords:** gene, CEUS, CXCR7, microvessel density

## Abstract

**Background:** The purpose of this study was to clarify the effect of C-X-C chemokine receptor type 7 (CXCR7) on proliferation, migration, and angiogenesis by changing the expression levels of CXCR7 in colon cancer cells. Contrast-enhanced ultrasound technology was used to quantify tumor perfusion parameters *in vivo* for the detection of angiogenesis after the change of CXCR7 expression in colon cancer xenografts.

**Methods:** To detect the expression of CXCR7 in colon cancer cells after overexpression or silencing of CXCR7. In addition, proliferation, migration, and angiogenesis were determined. The region of interest of the tumor was selected, and a time-intensity curve was drawn. Immunohistochemical staining was performed on tumor tissue sections, and the average microvessel density value was calculated.

**Results:** Overexpression or silencing of CXCR7 altered the proliferation, migration, and luminal formation of Caco-2 and SW480 cells. In xenografts produced using CXCR7-overexpressing or -silent Caco-2 and SW480, respectively, the peak intensity and area under the curve were significantly different. The expression of CXCR7, VEGF, Ki67, and CD34 was decreased in CXCR7-silent cells, but increased in CXCR7-overexpressing cells. CXCR7 apparently affected angiogenesis through the extracellular signal regulated kinase pathway.

**Conclusions:** The regulation of CXCR7 expression may affect the proliferation, migration, and luminal formation of Caco-2 and SW480 cells, indicating that CXCR7 may play an important role in colon cancer. Examination through contrast-enhanced ultrasound also demonstrated that the expression of CXCR7 is closely related to angiogenesis.

## Background

C-X-C chemokine receptor type 4 (CXCR4) was previously thought to be the only receptor for C-X-C motif chemokine ligand 12 (CXCL12). However, since the discovery of CXCR7^1^, researchers have begun to further review the mechanism of action of CXCL12. It has been proved that CXCR7 is not only expressed in tumor cells, but also expressed in normal cells^2^,^3^. Studies have shown that the the CXCL12/CXCR7 axis can promote tumor growth, migration, and angiogenesis^4^. Li^5^ showed that lipopolysaccharide stimulation induced the expression of CXCR7 in gastric cancer through toll-like receptor 4/myeloid differentiation factor 2 (TLR4/MD-2) signaling, thereby promoting the proliferation and migration of SGC7901 cells. Of particular note is the low CXCR7 expression in normal mature vascular endothelial cells, and the high expression in tumor endothelial cells^6^. Maishi^7^ suggested that CXCR7 can be used as a new vascular tumor endothelial marker. Tumor angiogenesis is considered to be the target of gene therapy and is closely related to tumor growth and metastasis^8^.

Contrast-enhanced ultrasound (CEUS) is a rapid and easy assessment method. It can be repeatedly performed in patients, and has high spatial and temporal resolution, providing an excellent opportunity for the visualization of microcirculation. It can display the shape and distribution of tumor blood vessels in real time, and be used to evaluate tumor angiogenesis. The use of CEUS to quantify tumor perfusion for the early detection of tumor angiogenesis has been previously described in the literature^9^-^11^. This study used the advantages of CEUS in microvascular imaging to detect angiogenesis in transplanted tumor tissues with overexpressed or silenced CXCR7 *in vivo*. Wang^12^ studied CEUS angiography, showing that the CEUS peak intensity (PI) and MVD of hepatocellular carcinoma were higher than those of peripheral tissues. Moreover, the PI was significantly correlated with MVD. Thus, quantitative analysis of CEUS parameters can contribute to the assessment of blood vessels in this disease. The relationship between CEUS angiographic parameters and MVD has also been studied in ovarian tumors, showing that the PI and AUC (area under the curve) are positively correlated with MVD in the benign and malignant groups^13^. In addition, research showed that the three-dimensional CEUS angiographic score was significantly associated with MVD and VEGF markers in 40 samples of human breast cancer before and after neoadjuvant chemotherapy^14^. Based on the above findings, we investigated the changes in the proliferation, migration, and luminal formation of CXCR7-overexpressing or -silent colon cancer cells. Also, CEUS was utilized for microvascular imaging to observe the effect of CXCR7 on angiogenesis.

## Material and Methods

### Cell culture

Caco-2 and SW480 colon cancer cell lines and human umbilical vein endothelial cells (HUVECs) were purchased from the Cell Bank of the Chinese Academy of Sciences (Shanghai, China). Caco-2 and SW480 cell lines were maintained in DMEM (HyClone, GE Healthcare, UK) with 10% fetal bovine serum (FBS, HyClone). The HUVECs were maintained in PRMI 1640 medium (HyClone) containing 10% fetal bovine serum (FBS, HyClone). All cells were cultured at 37°C and 5% CO2 in a humidified incubator.

### Cell transfection

For the CXCR7-overexpressing colon cancer group and its negative control (NC) group, Caco-2 cells were transfected with the pCMV6 Entry-CXCR7 plasmid (Caco-2^CXCR7OE^) and pCMV6 Entry vector (Caco-2^CXCR7NC^) (OriGene, WuXi, China), respectively. For the CXCR7-silent colon cancer group and its NC group, SW480 cells were transfected with short hairpin RNA (shRNA) (SW480^shRNACXCR7^) or unrelated sequences (SW480^shRNANC^) (RiboBio, Guangzhou, China), respectively. SW480 and Caco-2 cells without any treatment (Control). All cells were transfected with Lipofectamine 3000 (Invitrogen, Grand Island, NY, USA) and screened for stably transfected cells with G418 (Sigma-Aldrich, St Louis, MO, USA). The cells were subsequently cultured in an incubator with 5% CO2 at 37°C. The shRNA and control shRNA sequences are as follows: CXCR7: shRNA, 5′-GATCCCCGAGCTCACGTGCAAAGTCATTCAAGAGATGACTTTGCACGTGAGCTCTTTTT‐3′ (sense) and 5′‐AGCTAAAAAGAGCTCACGTGCAAAGTCATCTCTTGAATGACTTTGCACGTGAGCTCGGG‐3′ (antisense). NC: 5′‐GATCCCCTTCTCCGAACGTGTCACGTTTCAAGAGAACGTGACACGTTCGGAGAATTTTT‐3′ (sense) and 5′‐AGCTAAAAATTCTCCGAACGTGTCACGTTCTCTTGAAACGTGACACGTTCGGAGAAGGG‐3′ (antisense).

### qRT-PCR

Used TRIzol reagent (Invitrogen) to extract the total RNA of the sample, and reverse transcribed in accordance with the instructions provided in the reverse transcription kit (TaKaRa, Dalian, China). The primers used in this experiment were as follows: CXCR7, 5'-TCTGCATCTCTTCGACTACTCA-3' (forward) and 5'-GTAGAGCAGGACGCTTTTGTT-3' (reverse); glyceraldehyde-3-phosphate dehydrogenase (GAPDH): 5'-GAAGGTGAAGGTCGGAGT-3' (forward) and 5'-GAAGATGGTGATGGGATTTC- 3' (reverse). The expression of mRNA was calculated based on the 2-ΔΔCt method.

### Western blotting

Total protein lysates were prepared using the radioimmunoprecipitation assay buffer (Beyotime, Shanghai, China) for 15 min. Used the bicinchoninic acid protein concentration assay kit (Beyotime) to calculate the protein concentration of the sample. The dilution ratio of the anti-CXCR7 anti-GAPDH antibodies (Abcam, Cambridge, United Kingdom) was 1:1,000 and 1:2,000, respectively. The cells were incubated with the primary antibodies overnight at 4°C, followed by incubation with horseradish peroxidase-conjugated secondary antibodies (Abcam) (1:2,000 dilution). Visualization was achieved through chemiluminescence.

### CCK-8 and EdU to detect cell proliferation

Each group of Caco-2 or SW480 cells was seeded in 96-well plates with 3,000 cells per well. After culturing for 24, 48, and 72 h, 100 μL of medium containing 10% CCK-8 (Dojindo, Kumamoto, Japan) was added to each well at 37°C for 2 h, and the absorbance was measured at 450 nm (Thermo Fisher Scientific, Waltham, MA, USA). Prepared an appropriate amount of 50 μM EdU medium, and added 200 μL of medium to each well for 2 hours. The wells were subjected to Apollo staining in accordance with the instructions provided by the manufacturer, and photographed using a fluorescence microscope (Nikon,Japan).

### Cell migration assays

The transfected cells were seeded into the upper chamber with 4×10^5^cells per well. Subsequently, 500 μL of medium containing 20% FBS was added to the lower chamber, and the cells were incubated at 37°C for 6 h. A microscope (Nikon) was used to count the number of cells under the membrane surface in five different fields of view at ×200 magnification after fixation and staining.

### *In vitro* coculture assay for tube formation

The Matrigel™ tube formation assay was used to measure the ability of endothelial cells to arrange into tubular structures. Matrigel™ (200 μL) (BD Biosciences, San Diego, CA, USA) was spread in the lower chamber and incubated at 37°C for 30 min. Subsequently, the transfected cells (4×10^5^cells/well) were seeded in the upper chamber, while human umbilical vein endothelial cells (HUVEC; 2×10^5^cells/well) were seeded in the lower chamber coated with Matrigel™. After incubation at 37°C for 6 hours, the tube formation was counted and photographed with a microscope (Nikon).

### Establishment of a tumor model

Female nude mice, aged 4-6 weeks (Beijing Vital River Laboratory Animal Technology Co., Ltd., Beijing, China) were purchased and raised in a specific-pathogen-free environment. Each nude mouse (nine animals per group) was subcutaneously injected into the right lower extremity with 0.2 mL of a cell suspension containing 1×10^7^cells (Caco-2^CXCR7OE^, Caco-2^CXCR7NC^, SW480^shRNACXCR7^, or SW480^shRNANC^) in FBS. The mice were maintained for 4-6 weeks. CEUS examination was performed following tumor growth to approximately 1 cm^3^. After CEUS examinations, the mice were euthanized by CO2 inhalation. Animal studies were in compliance with the Guide for the Care and Use of Laboratory Animal Resources (1996), National Research Council and approved by the China Medical University Animal Ethics Committee (IACUC Issue No.16071). All procedures accepted the supervision and inspection by the committee and laboratory animal department.

### Conventional ultrasound and CEUS

Conventional two-dimensional ultrasound and CEUS were performed using the clinical diagnostic ultrasound system Aplio 500 (Toshiba Medical Systems, Co., Ltd., Ottowara, Japan) with a 10-MHz superficial probe. A specific calibration file (TCA, Toshiba Medical Systems) provided by the vendor was used as the analysis software. Ultrasound images were converted into linearized data for time-intensity curve (TIC) analysis. Firstly, the tumor size and morphology were carefully observed via two-dimensional ultrasound, and the color flow distribution was determined using color Doppler flow imaging. The acoustic contrast agent SonoVue (Bracco SpA, Milan, Italy) was used. The region of interest of the tumor was selected, and 100 µL of ultrasound contrast agent was injected into the tail vein of each mouse. Subsequently, the ultrasound contrast image and the original data were acquired. All ultrasound images were saved in a computer, and a TIC was constructed for the region of interest of the specified image using the system's own image analysis software. The following perfusion parameters were obtained: PI, time to peak (TTP), mean transit time (MTT), AUC, and slope.

### Histopathology and MVD analysis

After acquiring the CEUS images, each mouse was euthanized and the tumor tissue was removed. Tumor tissue sections corresponding to the ultrasound imaging plane were prepared for immunohistochemical staining. The sections were fixed in 10% formalin, embedded in paraffin, cut into slices (thickness: 4 µm), dewaxed with xylene, and immersed in 100%, 95%, 85%, and 75% alcohol for 5 min. The sections were quenched with 3% hydrogen peroxide for 10 min, and incubated for 10 min in protein blocking solution (1% goat serum in phosphate-buffered saline). They were subsequently incubated with primary antibody overnight at 4°C. The primary antibodies and dilution concentrations were as follows: CXCR7 antibody (1:200), CD34 antibody (1:2,500), ki67 antibody (1:100), VEGF antibody (1:100), extracellular signal regulated kinase (ERK) antibody (1:100), and p-ERK antibody (1:500) (Abcam). The blots were probed with antibodies specific for ERK phosphorylation at Thr202 and Tyr204. After addition of the secondary antibody (peroxidase-conjugated goat anti-rabbit IgG or peroxidase-conjugated goat anti-mouse IgG [Abcam]) and incubation for 2 h, diaminobenzidine was added dropwise for color development. Brown staining indicated positivity for the primary antibody. Immunohistochemical results were scored to determine the intensity of positive staining and the proportion of tumor cells.

MVD measurements (the gold standard for assessing tumor angiogenesis) were determined according to the quantitative method described by Weidner^15^. Firstly, the entire section was observed under a low-power microscope (Nikon) (magnification, x100), and three regions with the highest vessel density (hotspots) were selected. Thereafter, CD34-stained blood vessels (CD34 is an endothelial cell marker) were counted using a high-power microscope (Nikon) (magnification, x200), and the mean MVD value was calculated.

### Statistical analysis

Data analysis and processing were performed using the SPSS 19.0 (IBM Corp., Armonk, NY, USA) statistical software. One-way analysis of variance was used for comparison between groups. Spearman's rank correlation analysis of CEUS perfusion parameters and MVD was performed; p<0.05 denoted statistically significant difference.

## Results

### Expression levels of CXCR7 in colon cells

**Figure 1:**
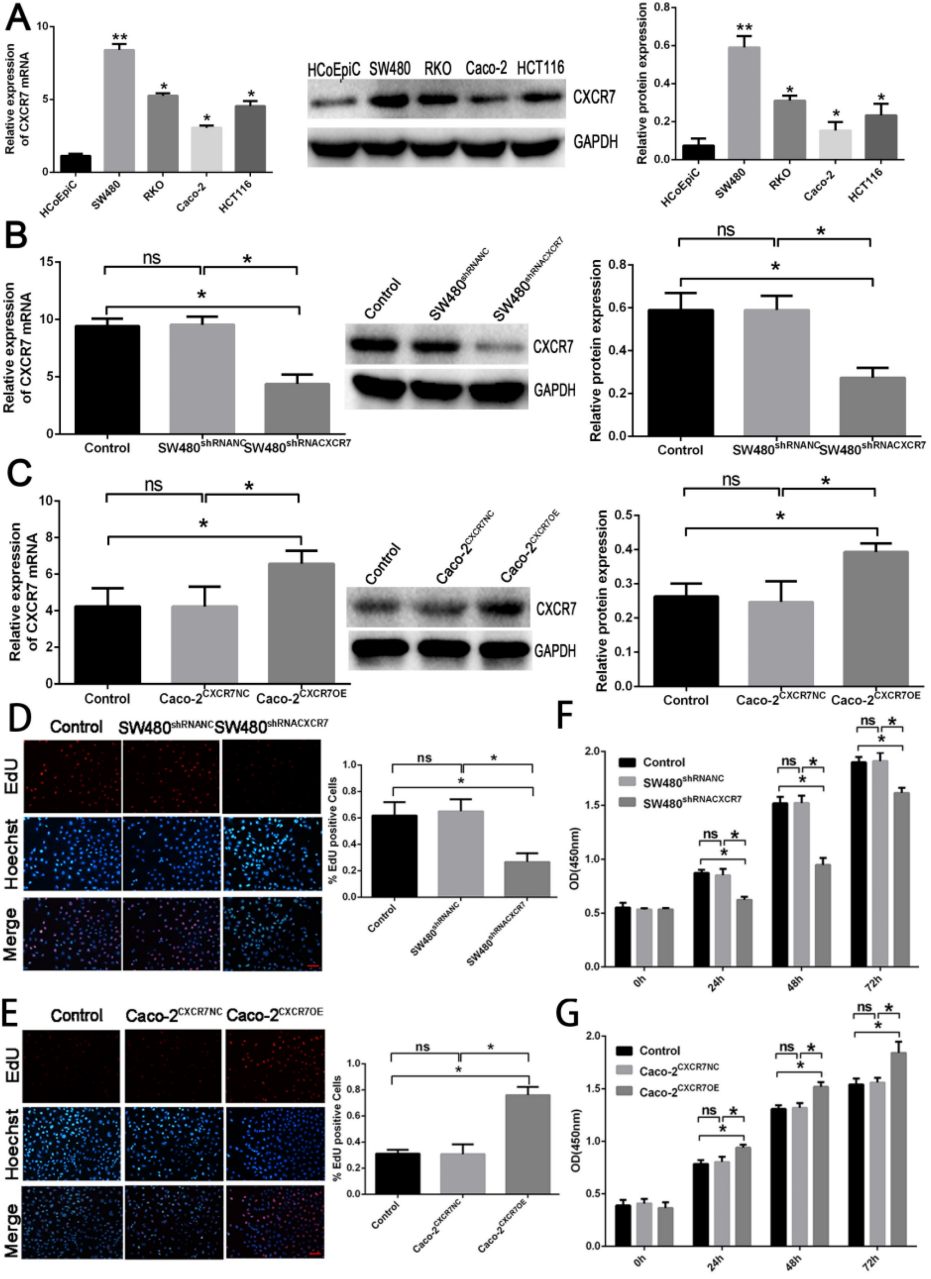
Effect of CXCR7 in Caco-2 and SW480 cells. (A) qRT-PCR and western blotting detection of mRNA and protein expression. (B) qRT-PCR and western blotting detection of mRNA and protein expression in CXCR7-silent SW480 cells. (C) qRT-PCR and western blotting detection of mRNA and protein expression in CXCR7-overexpressing Caco-2 cells. (D) Left panel: representative image of EdU-positive cells among CXCR7-silent SW480 cells. Right panel: quantitative measurement of EdU-positive cells among CXCR7-silent SW480 cells. (E) Left panel: representative image of EdU-positive cells among CXCR7-overexpressing Caco-2 cells. Right panel: Quantitative measurement of EdU-positive cells among CXCR7-overexpressing Caco-2 cells. (F) After transfection with CXCR7-silent SW480 cells for 0, 24, 48, and 72 h, the proliferative ability of CXCR7-silent SW480 cells was markedly reduced. (G) After transfection with CXCR7-overexpressing Caco-2 cells for 0, 24, 48, and 72 h, the proliferative ability of CXCR7-overexpressing Caco-2 cells was markedly increased. Magnification ×200. n = 3, and each experiment was repeated 3 times. *p<0.05; **p<0.01 vs. HCoEpic or NC groups.

The expression of CXCR7 in colon cancer cell lines (including HCoEpic, RKO, HCT116, SW480, and Caco-2) was evaluated by qRT-PCR and western blotting (Figure 1A). The expression of CXCR7 in the four colon cancer cell lines was significantly higher than that of normal colon cancer cells (HCoEpic). Among them, SW480 cells had the highest expression of CXCR7 and Caco-2 cells had the lowest expression.

### Expression levels of CXCR7 after transfection

The expression of CXCR7 in CXCR7-silent SW480 cells and CXCR7-overexpressing Caco-2 cells was detected by qRT-PCR and western blotting. The results showed that the protein expression and mRNA levels of CXCR7 in CXCR7-silent SW480 cells were markedly reduced compared with those of the NC group (Figure 1B). In contrast, the results showed that the protein expression and mRNA levels of CXCR7 in CXCR7-overexpressing Caco-2 cells were markedly increased compared with those of the NC group (Figure 1C).

### Effect of CXCR7 on the proliferation

We investigated the effect of CXCR7 overexpression or silencing on the proliferation of colon cancer cells. For this purpose, EdU and CCK-8 were used to detect the proliferative activity of CXCR7-overexpressing or -silent colon cancer cells. The number of EdU-positive cells among CXCR7-silent SW480 cells was markedly reduced. In contrast, the number of EdU-positive cells among CXCR7-overexpressing Caco-2 cells was markedly increased (Figures 1D and 1E). CXCR7-overexpressing or -silent colon cancer cells were transfected for 0, 24, 48, and 72 h. The results of the CCK-8 assay showed that the proliferative ability of CXCR7-silent SW480 cells was significantly decreased from 24 h. In contrast, the proliferative ability of CXCR7-overexpressing Caco-2 cells was significantly increased from 24h (Figures 1F and 1G).

### Effect of CXCR7 on the migration

**Figure 2:**
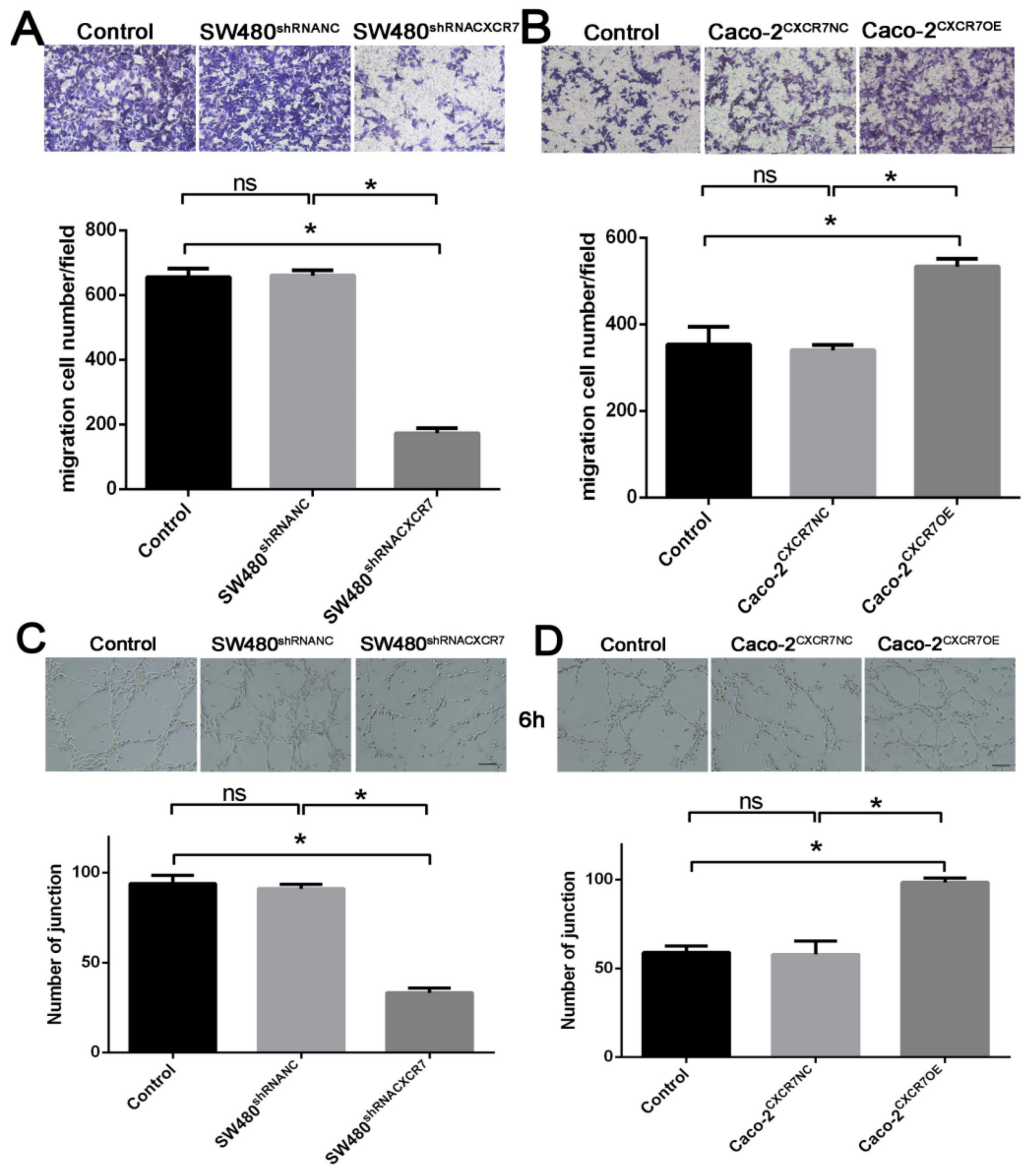
Effect of CXCR7 on the migration and formation of HUVEC lumen. (A) Upper panel: representative image of the migration of CXCR7-silent SW480 cells. Lower panel: quantitative measurement of the number of migrating cells among CXCR7-silent SW480 cells. (B) Upper panel: representative image of the migration of CXCR7-overexpressing Caco-2 cells. Lower panel: quantitative measurement of the number of migrating cells among CXCR7-overexpressing Caco-2 cells. (C) Upper panel: representative image of the lumen formation of HUVEC co-cultured with CXCR7-silent SW480 cells. Lower panel: quantitative measurement of the number of junctions among CXCR7-silent SW480 cells. (D) Upper panel: representative image of the lumen formation of HUVEC co-cultured with CXCR7-overexpressing Caco-2 cells. Lower panel: quantitative measurement of the number of junctions among CXCR7-overexpressing Caco-2 cells. Magnification ×200. n = 3, and each experiment was repeated 3 times. *p<0.05 vs. NC groups.

The results of the Transwell migration experiment showed that the migratory ability of CXCR7-silent SW480 cells was lower than in the NC group (Figure 2A). On the contrary, the migratory ability of CXCR7-overexpressing Caco-2 cells was higher than in the NC group (Figure 2B).

### Effect of co-culture with CXCR7-overexpressing or -silent colon cancer cells on the formation of HUVEC lumen

CXCR7-silent SW480 cells and CXCR7-overexpressing Caco-2 cells were co-cultured with HUVEC. The lumen-forming ability of HUVEC on Matrigel was evaluated by measuring the number of junctions. After co-culture with CXCR7-silent SW480 cells, the angiogenic ability of HUVEC was markedly reduced (Figure 2C). On the contrary, after co-culture with CXCR7-overexpressing Caco-2 cells, the angiogenic ability of HUVEC was markedly enhanced (Figure 2D).

### CEUS-based observations of tumor microcirculation

**Figure 3:**
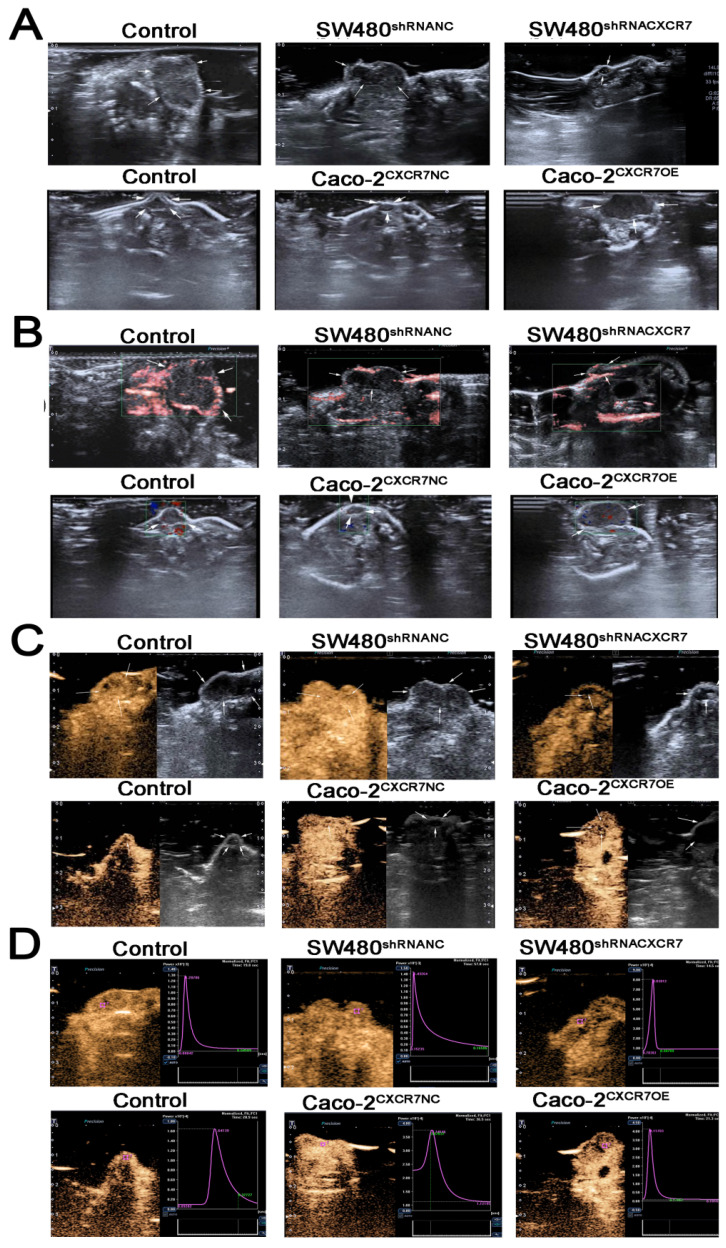
CEUS-based observations of tumor microcirculation. (A) Two-dimensional ultrasound of a xenograft tumor model. (B) Color Doppler flow imaging (CDFI). (C) CEUS angiography. (D) Tumor time-intensity curve (TIC).

The examination using two-dimensional ultrasound showed that the transplanted tumors were generally elliptical or irregular, and their boundaries were clear. The interior had a medium or low echo, and the distribution was not uniform (Figure 3A). The color Doppler flow imaging results showed that blood flow signals were visible around the tumor, whereas the blood flow signal inside the tumor was lower or the visualization was unclear (Figure 3B). After CEUS, the number of blood vessels was increased, the length was increased, and the blood flow display ability was significantly improved. Thus, information regarding the blood flow was increased compared with that obtained from the examination using two-dimensional ultrasound. Dynamic videos showed that blood vessels permeated from around the tumor to its interior, showing a more complete network of tumor blood vessels (Figure 3C). The following perfusion parameters were obtained by analyzing the TIC after angiography: PI, TTP, MTT, AUC, and slope (Figure 3D). In the CXCR7-silent SW480 xenografts, the PI, MTT, slope, and AUC were decreased. However, the TTP was elevated. The differences in the PI, TTP, MTT, slope and AUC were significant; of note, differences for the PI and AUC were highly significant (p<0.001). In the CXCR7-overexpressing Caco-2 xenografts, the PI, slope, and AUC were increased compared with those recorded in the NC group. However, the MTT and TTP were decreased. The differences in the PI, MTT, and AUC were significant; of note, differences for the PI and AUC were highly significant (p<0.001) (Table 1).

### Proliferation, angiogenesis, and ERK pathway analysis

**Figure 4:**
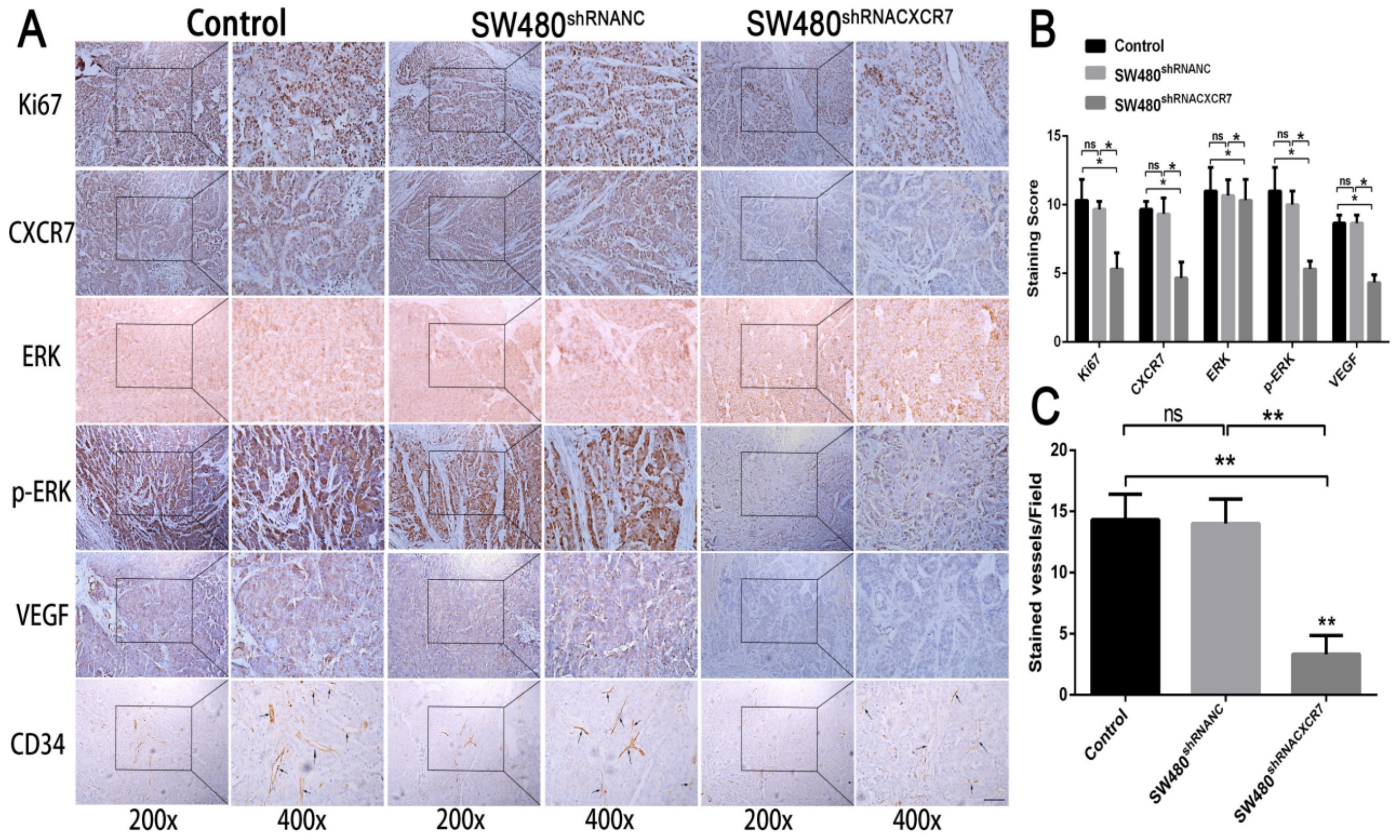
Immunohistochemical analysis in SW480 colon cancer tissue. (A) Subcutaneous tumors with different levels of CXCR7 expression were harvested and immunohistochemically stained for CXCR7, VEGF, Ki67, ERK, p-ERK, and CD34. (B) Immunohistochemical staining score results. (C) Microvessel density (MVD) indicating a marked reduction in microvessels in CXCR7-silent SW480 tissues. Magnification ×200 and ×400. n = 3, and each experiment was repeated 3 times. *p<0.05 vs. NC groups.

Immunohistochemical staining for CXCR7, VEGF, Ki67, and CD34 was reduced in the CXCR7-silent SW480 group. We further investigated the role of the ERK pathway in angiogenesis. The results showed that antibody staining of p-ERK in the CXCR7-silent group was significantly reduced with lower staining scores (Figure 4A and 4B).

**Figure 5:**
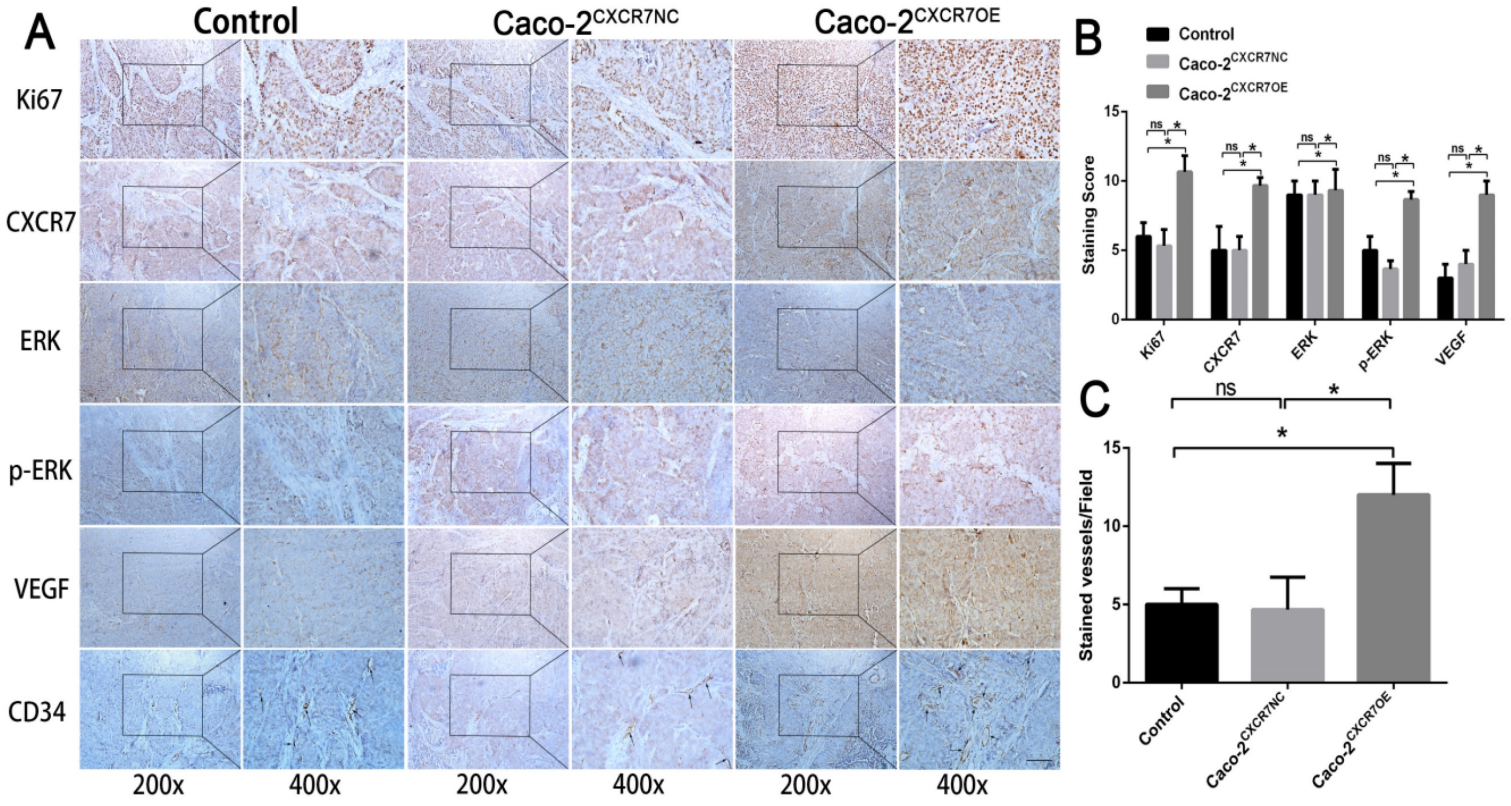
Immunohistochemical analysis in Caco-2 colon cancer tissue. (A) Subcutaneous tumors with different levels of CXCR7 expression were harvested and immunohistochemically stained for CXCR7, VEGF, Ki67, ERK, p-ERK, and CD34. (B) Immunohistochemical staining score results. (C)MVD indicating a marked increase in microvessels in CXCR7-overexpressing Caco-2 tissues. Magnification ×200 and ×400. n = 3, and each experiment was repeated 3 times. *p<0.05 vs. NC groups.

In contrast, immunohistochemical staining for CXCR7, VEGF, Ki67, and CD34 was increased in the CXCR7-overexpressing Caco-2 cells group. Antibody staining of p-ERK after CXCR7 overexpression was significantly increased with higher staining scores (Figure 5A and 5B). We examined the tumor MVD using CD34 immunohistochemistry, and the results showed that the MVD in the CXCR7-silent group was reduced. In contrast, the MVD in the CXCR7-overexpressing group was increased (Figure 4C and 5C).

The expression of VEGF, ERK, and p-ERK in CXCR7-silent SW480 cells and CXCR7-overexpressing Caco-2 cells was detected by western blotting. The results showed that the protein expression of VEGF and p-ERK in CXCR7-silenced SW480 cells were markedly reduced (Figure S1A). In contrast, the protein expression of VEGF and p-ERK in CXCR7-overexpressing Caco-2 cells were markedly increased (Figure S1B).

### Correlation between perfusion parameters and MVD

Next, we investigated the relationships between the perfusion parameters and MVD in tissues with silenced and overexpressed CXCR7 (Table 2). In SW480 and Caco-2 tissues, the PI and AUC were correlated with MVD, whereas TTP, MTT, and slope were not. The PI and AUC were less strongly correlated with MVD in the CXCR7-silent SW480 cells (PI: r = 0.323, p<0.05; AUC: r = 0.237, p<0.01) than in the NC group (PI: r = 0.736, p<0.05; AUC: r = 0.698, p<0.05). The PI and AUC were more strongly correlated with MVD in the CXCR7-overexpressing Caco-2 cells (PI: r = 0.798, p<0.05; AUC: r = 0.802, p<0.05) than in the NC group (PI: r = 0.513, p<0.05; AUC: r = 0.447, p<0.05).

## Discussion

A growing body of evidence indicates that CXCR7 may be involved in tumor development. CXCR7 was highly expressed in various tumor cells^16^,^17^. Moreover, the expression of CXCR7 promotes the proliferative, migratory, and angiogenic abilities of tumor cells *in vitro*, and promotes tumor growth *in vivo*^18^,^19^. In this study, we found that the expression of CXCR7 was increased in all four colon cancer cell lines compared with normal colon cells. Among them, SW480 cells and Caco-2 cells were screened for silencing and overexpression of CXCR7, and their proliferative and migratory abilities were tested. The results showed that changes in CXCR7 expression can affect the proliferation and migration. Ma^20^ found that the interaction of SDF-1 / CXCR7 participates in the angiogenesis of gastric cancer by promoting the secretion of VEGF, and knocking down CXCR7 could play an inhibitory effect. We have observed that colon cancer cells can induce luminal formation *in vitro*, thereby promoting tumor growth.

CEUS is a major development in ultrasonography^21^. In traditional Doppler ultrasound, small blood vessels in tumors are unclear, particularly those with low flow rates. However, when an ultrasound contrast agent is used, the agent remains in the blood vessel for a period of time and the backscattered sound intensity of the microbubble is linearly related to the concentration^22^. CEUS allows the continuous and dynamic observation of tumor vascular perfusion^23^. CEUS can display capillary blood flow in real time; hence, subtle changes in tumor microcirculation perfusion can be detected. In this study, we used CEUS to detect the perfusion of CXCR7 overexpression and silencing in colon tumors. This approach can visually show the angiogenesis in mouse colon cancer tumors after changes in the expression of CXCR7. MVD is an independent prognostic indicator that has been recognized as the gold standard for assessing tumor angiogenesis^24^. The accuracy of CEUS regarding angiogenesis was confirmed by a correlation analysis between CEUS parameters and MVD (based on CD34 immunohistochemistry). The results also showed that, in the CXCR7-silent colon cancer tissues, the perfusion parameters PI and AUC were reduced. In contrast, in the CXCR7-overexpressing colon cancer tissues, the PI and AUC were increased.

Previous studies have shown that the ERK pathway is important for tumor survival, proliferation, and angiogenesis^25^,^26^. This study further examined the expression of Ki67, VEGF, ERK, and p-ERK. The results showed that changes in CXCR7 expression affected the phosphorylation of ERK, and altered the expression of Ki67 and VEGF. Additionally, CD34 was positively correlated with CXCR7 expression. This suggests that CXCR7 promotes colon cancer angiogenesis via the ERK pathway, and the results provide a preliminary theoretical basis for future research. The regulation of CXCR7 expression may affect the proliferation, migration, and luminal formation of Caco-2 and SW480 cells. The CEUS parameters in this study, especially the PI and AUC, were closely related to MVD. The results also suggested that CXCR7 is directly involved in angiogenesis, and its role in tumor angiogenesis can be assessed by detecting tumor perfusion changes through quantitative CEUS.

## Supplementary Material

Supplementary figure.

## Figures and Tables

**Table 1:** Comparison of the tumor CEUS parameters in SW480 and Caco-2 colon cancer tissue (mean±SD)

Group	PI (10E-5 AU)	TTP (s)	MTT (s)	Slope (10E-5 AU/s)	AUC (10E-5 AU.s)
**Control**	120.4±12.5	1.3±0.8	3.8±1.5	257.5±71.0	1129.0±64.4
**SW480^shRNANC^**	117.2±11.9	1.2±0.6	3.6±1.0	244.2±32.2	1184.7±87.6
**SW480^shRNACXCR7^**	65.0±7.4***	2.6±1.0	2.2±1.0	31.3±10.2	612.4±58.0***
**Control**	17.1±3.4	2.2±1.6	5.7±1.5	16.7±8.3	102.5±5.8
**Caco-2^CXCR7NC^**	14.7±0.8	3.3±1.3	5.7±0.8	18.7±2.7	102.9±9.9
**Caco-2^CXCR7OE^**	44.1±4.6***	1.6±1.2	2.6±1.4	26.3±12.0	210.9±10.1***

**Notes:** The perfusion parameters PI and AUC were reduced in the CXCR7-silent SW480 transplanted tumor tissue compared with the NC group. The perfusion parameters PI and AUC were increased in the CXCR7-overexpressing Caco-2 transplanted tumor tissue compared with the NC group. ***p<0.001 vs. NC group.

**Table 2:** Correlation between tumor CEUS parameters and microvessel density (MVD) in SW480 and Caco-2 colon cancer tissues (mean±SD)

CEUS parameters	Control r, p	SW480^shRNANC^ r, p	SW480^shRNACXCR7^ r, p	Control r, p	Caco-2^CXCR7NC^ r, p	Caco-2^CXCR7OE^ r, p
**PI (10E-5 AU)**	0.834	0.736	0.323	0.482	0.513	0.798
	0.026*	0.032*	0.045*	0.046*	0.038*	0.016*
**TTP (s)**	-0.236	-0.154	-0.042	-0.021	-0.128	-0.215
	0.051	0.626	0.428	0.551	0.068	0.626
**MTT (s)**	-0.032	-0.058	-0.362	-0.059	-0.145	-0.029
	0.094	0.725	0.238	0.095	0.327	0.485
**Slope (10E-5 AU/s)**	0.167	0.047	0.027	0.175	0.127	0.044
	0.487	0.229	0.096	0.571	0.692	0.357
**AUC (10E-5 AU.s)**	0.758	0.698	0.237	0.451	0.447	0.802
	0.012*	0.035*	0.006**	0.012*	0.024*	0.017*

**Notes:** The PI and AUC were less correlated with MVD in CXCR7-silent SW480 cells; on the contrary, they were more correlated with MVD in CXCR7-overexpressing Caco-2 cells. *p<0.05, **p<0.01 vs. NC group.

## Abbreviations

CEUS: contrast-enhanced ultrasound
CXCR7: C-X-C chemokine receptor type 7
VEGF: vascular endothelial growth factor
TTP: time to peak
AUC: area under the curve
MTT: mean transit time
PI: peak intensity
shRNA: short hairpin RNA
qRT-PCR: quantitative reverse-transcription polymerase chain reaction
CCK-8: Cell Counting Kit-8
EdU: 5-ethynyl-20-deoxyuridine
OD: optical density
MVD: microvessel density
