# Supplementary material for: Contrast-enhanced ultrasound for the evaluation of CXCR7-mediated angiogenesis in colon cancer
Source: J Cancer. 2023 Mar 5;14(4):665–75. doi: 10.7150/jca.82438 (PMC10088541; doi:10.7150/jca.82438)
Supplement: Supplementary file 1 — Supplementary figure. [file jcav14p0665s1.pdf]

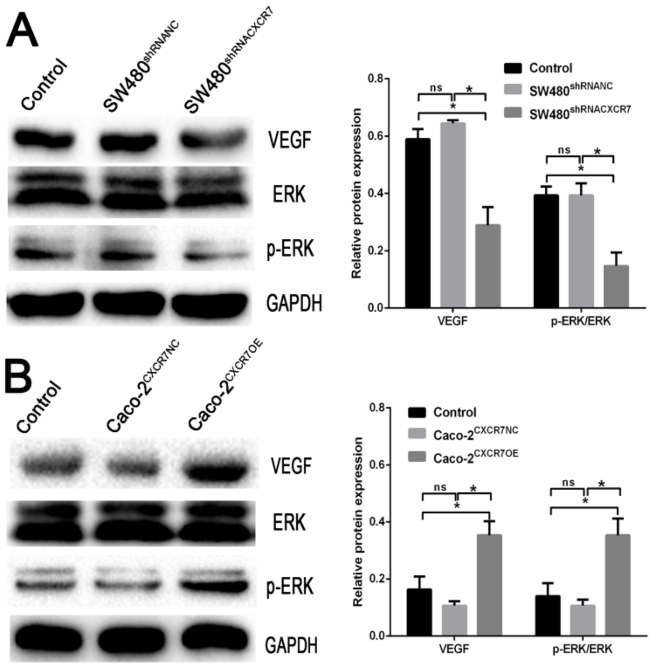

Fig S1 Effects of CXCR7 on VEGF and ERK pathway expression.(A) Western blotting detection of protein expression in CXCR7-silent SW480 cells. (B) Western blotting detection of protein expression in CXCR7-overexpressing Caco-2 cells. n = 3, and each experiment was repeated 3 times. *p<0.05 vs. NC groups.
